# Saponin-Derived Silver Nanoparticles from *Phoenix dactylifera* (Ajwa Dates) Exhibit Broad-Spectrum Bioactivities Combating Bacterial Infections

**DOI:** 10.3390/antibiotics12091415

**Published:** 2023-09-07

**Authors:** Mohd Adnan, Arif Jamal Siddiqui, Syed Amir Ashraf, Mohammad Saquib Ashraf, Sarah Owdah Alomrani, Mousa Alreshidi, Bektas Tepe, Manojkumar Sachidanandan, Corina Danciu, Mitesh Patel

**Affiliations:** 1 Department of Biology, College of Science, University of Ha’il, Ha’il 55473, Saudi Arabia; drmohdadnan@gmail.com (M.A.);; 2 Medical and Diagnostic Research Centre, University of Ha’il, Ha’il 55473, Saudi Arabia; 3 Department of Clinical Nutrition, College of Applied Medial Sciences, University of Ha’il, Ha’il 55473, Saudi Arabia; 4 Department of Medical Laboratory Science, College of Applied Medical Sciences, Riyadh ELM University, Riyadh 12734, Saudi Arabia; 5 Department of Biology, College of Science and Arts, Najran University, Najran 66252, Saudi Arabia; 6 Department of Molecular Biology and Genetics, Faculty of Science and Literature, Kilis 7 Aralik University, TR-79000 Kilis, Turkey; 7 Department of Oral Radiology, College of Dentistry, University of Ha’il, Ha’il 55473, Saudi Arabia; 8 Department of Pharmacognosy, Faculty of Pharmacy, “Victor Babes” University of Medicine and Pharmacy, 2 Eftimie Murgu Square, 300041 Timisoara, Romania; 9 Research and Development Cell, Department of Biotechnology, Parul Institute of Applied Sciences, Parul University, Vadodara 391760, India

**Keywords:** antibiofilm, anti-quorum sensing, cytotoxicity, antibacterial, antibiotic resistance, saponins, *Phoenix dactylifera*, Ajwa date

## Abstract

The emergence of antibiotic resistance poses a serious threat to humankind, emphasizing the need for alternative antimicrobial agents. This study focuses on investigating the antibacterial, antibiofilm, and anti-quorum-sensing (anti-QS) activities of saponin-derived silver nanoparticles (AgNPs-S) obtained from Ajwa dates (*Phoenix dactylifera* L.). The design and synthesis of these novel nanoparticles were explored in the context of developing alternative strategies to combat bacterial infections. The Ajwa date saponin extract was used as a reducing and stabilizing agent to synthesize AgNPs-S, which was characterized using various analytical techniques, including UV–Vis spectroscopy, Fourier transform infrared (FTIR) spectroscopy, and transmission electron microscopy (TEM). The biosynthesized AgNPs-S exhibited potent antibacterial activity against both Gram-positive and Gram-negative bacteria due to their capability to disrupt bacterial cell membranes and the leakage of nucleic acid and protein contents. The AgNPs-S effectively inhibited biofilm formation and quorum-sensing (QS) activity by interfering with QS signaling molecules, which play a pivotal role in bacterial virulence and pathogenicity. Furthermore, the AgNPs-S demonstrated significant antioxidant activity against 2,2-diphenyl-1-picrylhydrazyl (DPPH) free radicals and cytotoxicity against small lung cancer cells (A549 cells). Overall, the findings of the present study provide valuable insights into the potential use of these nanoparticles as alternative therapeutic agents for the design and development of novel antibiotics. Further investigations are warranted to elucidate the possible mechanism involved and safety concerns when it is used in vivo, paving the way for future therapeutic applications in combating bacterial infections and overcoming antibiotic resistance.

## 1. Introduction

Nanotechnology is a rapidly advancing area that deals with the manipulation and control of matter at the nanoscale level, typically involving structures with dimensions ranging from 1 to 100 nanometers [1]. At this scale, materials frequently display distinctive qualities and behaviors that are different from those of their bulk counterparts [2]. Nanotechnology has attracted significant attention due to its potential to revolutionize various industries and offer solutions to numerous challenges in many disciplines, such as pharmaceutical industries, electronics, energy, and environmental science [3,4,5]. One particular area of interest within nanotechnology is the utilization of nanoparticles, which are tiny particles with dimensions on the nanoscale [6,7]. Among the several kinds of nanoparticles, silver nanoparticles (AgNPs) have attracted a great deal of interest and have emerged as a prominent nanomaterial with diverse applications [8]. AgNPs have unique chemical, physical, and biological characteristics that make them highly versatile for a variety of applications [9,10]. The unique properties of AgNPs, such as their high surface-area-to-volume ratio, high electrical and thermal conductivity, and antimicrobial properties, contribute to their extensive applications in many disciplines such as the pharmaceutical industries, electronics, environmental science, and consumer products [11,12,13]. These characteristics make AgNPs versatile and attractive for use in relation to diverse technological advancements, paving the way for exciting developments in the nanotechnology arena [14].

The drugs previously used to inhibit or kill viruses, bacteria, and fungi have become less effective over time [15]. It has been found that the improper use and misuse of antimicrobials can lead to resistance in bacteria. This is particularly problematic in developing nations where patients can obtain antibiotics without a medical prescription [16]. Several antibiotics that are designed to inhibit the growth of bacteria and fungi are being phased out due to the fact that the target microorganisms are becoming resistant to them. Thus, antibiofilm effects and QS inhibition are currently regarded as two new methods that can be employed to tackle microbial resistance, thereby alleviating the ferocity of infections associated with them [17]. There are many kinds of bacteria that produce an extracellular polymeric protective coating, referred to as a biofilm, on abiotic or biotic surfaces. These coatings are most commonly found on surfaces with adequate nutrients or on food sources, and they protect bacteria against antibiotics, disinfectants, detergents, and defense systems that occur in the host body [18]. The process of QS involves the synthesis, diffusion, detection, and reaction of small signaling molecules known as autoinducers that signal to other cells. In fact, biofilm formation is a result of QS, which increases the resistance of bacteria to antibiotics 10–1000 times more than in their planktonic stage [19]. By disrupting the biofilm formation and QS in bacteria, it is possible to limit the expression of virulence factors in pathogenic bacteria, as well as the infection and contamination of such microorganisms.

It is widely recognized that natural foodstuffs such as fruits can provide human bodies with nutrients and a range of bioactive molecules that contribute to a healthy body. As strategic antimicrobials, these natural products are being looked into as potential food additives or nutraceuticals, which can serve as nutritional supplements for the good health of consumers. The inhibition of the production of signal molecules aided by synthesized signal molecules is a process called QS inhibition, which can be used to target cell-to-cell communication. When the production of the signal molecules is blocked, the bacteria are not able to form biofilms [20]. As a result, most scientists are currently working on the development of new therapeutic antibiotics by investigating plant products in order to find new antibiotics, which prevent the development of resistant bacteria strains by inhibiting QS mechanisms and controlling infections [21].

There is enormous interest in plants being used to synthesize different types of metal nanoparticles. Using plant metabolites in the synthesis of AgNPs is advantageous due to their natural origins and low toxicity [22]. In recent years, several reports have been published on the process by which plant-mediated AgNPs are synthesized. The results of these studies have shown that plant metabolites can be used to synthesize bioactive AgNPs with high efficiency. There are different types of secondary metabolites that can be found in plants, such as flavonoids, phenolic compounds, terpenoids, and saponins, which have been reported to play a role in reducing silver ions to their elemental state [23]. Due to the fact that plant species vary in terms of their composition and the quantity of secondary metabolites, it is important for us to examine a broad range of plant species to determine their ability and efficiency in biosynthesizing metal nanoparticles [24]. In this way, biomedical constraints and other production-related issues can be overcome. By using phyto-molecules to synthesize AgNPs, we may be able to avoid the problems associated with toxic chemical reagents in the production of AgNPs [25].

Ajwa dates (*Phoenix dactylifera* L.) are an expensive and popular fruit limited to Saudi Arabia’s holy city of Madinah Al Munawara and its surrounding areas [26]. There is an abundance of dietary fiber in Ajwa dates, which may help to solve digestion problems. By providing natural roughage to the body and stimulating bowel movements, Ajwa dates effectively relieve constipation [27]. Furthermore, these dates contain large amounts of potassium, which is necessary for muscle contractions. Ajwa dates enrich breast milk with many nutrients that are beneficial for lactating women. Ajwa dates have also been shown to reduce disease and infection susceptibility in the children of mothers who eat them regularly [28]. Additionally, Ajwa dates are high in iron, which is another significant advantage. Besides assisting in the production of red blood cells, iron may also assist in treating and preventing anemia. As a result of their nutritional and health properties, Ajwa dates can be thought of as a potential bioactive component for the development of food products with nutraceutical significance for various health purposes [29]. Ajwa dates usually accumulate polyphenols, triterpenoids, saponins, and flavonoids, which confer some medicinal properties, including protection against cardiovascular disorders, diabetes, and cancer. Furthermore, their antimicrobial, anti-inflammatory, and antioxidant properties suggest their potential benefits in supporting immune function and reducing the risk of various infections [30].

The aim of this study was therefore to extract and synthesize saponin-derived AgNPs from Ajwa dates (AgNPs-S). Different biophysical methods were used to characterize the formation of AgNPs-S. Furthermore, the synthesized AgNPs-S were also tested against several bacterial pathogens to determine their antibacterial, nucleic acid, and protein leakage and their anti-QS and antibiofilm properties. The production of violacein, pyocyanin, and prodigiosin, which is considered a QS phenomenon, was examined in the presence of AgNPs-S. Additionally, the antioxidant activity of AgNPs-S against DPPH free radicals and their cytotoxic potential against human small lung cancer cells (A549s) were further assessed.

## 2. Materials and Methods

### 2.1. Collection of Ajwa Dates

The Ajwa dates used in the present study were purchased fresh from Al-Madina Al-Munawwarah, the Kingdom of Saudi Arabia. After manual separation, the pulpy part of the date fruits was washed with double-distilled water, dried in an oven, and coarsely powdered using an electrical grinder. After the coarse powder was prepared for experimentation, it was kept in an airtight jar.

### 2.2. Extraction of Crude Saponins

In order to extract crude saponins from the Ajwa dates, powdered samples (20 g) were heated at 55 °C for 4 h with 100 mL of ethanol (20%). After the extract was filtered, residues were re-extracted using 200 mL of ethanol (20%). The extract was concentrated to 40 mL in a water bath and then combined with 20 mL of diethyl ether in a separating funnel. The mixture was vigorously agitated to separate the diethyl and aqueous layers. The aqueous phase was collected, and the diethyl ether fraction was discarded. N-butanol (60 mL) was added and thoroughly mixed in the aqueous layer. In the next step, 10 mL of 5% NaCl solution was added to the n-butanol extract. A water bath was used to concentrate the solution, and the saponin residues were dried in an oven [31].

### 2.3. Foam Test for Saponins

Saponins were diluted with 20 mL deionized water; then, samples were shaken for 15 min. The presence of saponins was suggested by the development of a stable foam.

### 2.4. Spectrophotometric Analysis of Saponins

A spectrophotometric method was used, with minor modifications, to quantitate the saponin-enriched fractions [32]. Quil-A (QA) was used as a standard. A solution of p-anisaldehyde (Sigma Aldrich^®^, Bengaluru, India) in ethyl acetate (0.5:99.5) and a solution of H_2_SO_4_ were mixed in equal amounts. QA and saponins were mixed with 2 mL of ethyl acetate. In the next step, reagents A and B were added (1 mL) to the reaction mixture. An incubation at 60 °C was performed in a water bath for 10 min on the mixture. After cooling the solutions for 10 min, they were measured at 430 nm for absorbance. The absorbance was measured using ethyl acetate as a control. To obtain a calibration curve, 75–175 µg of standard saponin was mixed in 2 mL of ethyl acetate. The overall saponins in the extract were calculated using a calibration curve for standard saponin.

### 2.5. High-Performance Thin-Layer Chromatography (HPTLC)

The HPTLC technique was used to analyze saponin-rich fractions obtained from Ajwa dates. After pre-washing with methanol and activating at 100 °C for 30 min, a silica gel 60 F_254_ plate (20 × 10 cm) was utilized for the chromatographic analysis. Separation was performed with chloroform, methanol, and water (6.4:3.2:1.2:0.8 *v*/*v*/*v*). Using an HPTLC autosampler (ATS4, CAMAG AG, Muttenz, Switzerland), a 10 µL sample was injected in 8 mm bands under a nitrogen flow (ATS4, CAMAG AG, Muttenz, Switzerland). HPTLC chambers were developed and presaturated with solvent systems and were used for separation until the migration of the solvent front reached 70 mm. In the following step, a vacuum-drying process was performed on the plates for 10 min [33].

### 2.6. Derivatization with p-Anisaldehyde Sulphuric Acid after Chromatography

The developed chromatographic plate was post-chromatographically derivatized by dipping it using a p-anisaldehyde sulphuric acid coloring solution (for 5 s). After drying for 10 min, a temperature of 70 °C was further applied to the plate for 5 min until bands appeared. A photo documentation system was used for the detection (TLC visualizer, CAMAG AG, Muttenz, Switzerland) of visible and ultraviolet light wavelengths. With a slit length of 5.00 × 0.45 mm and a wavelength of 545 nm, a densitometric measurement was performed (CAMAG AG, Muttenz, Switzerland). A densitogram was evaluated via winCats (CAMAG AG, Muttenz, Switzerland) [34].

### 2.7. Biosynthesis of Silver Nanoparticles Using Extracted Saponins (AgNPs-S)

The development of saponin-derived AgNPs was carried out by adding 4 mL of the extracted saponins drop by drop into 16 mL of 0.003 M aqueous silver nitrate and stirring it at 60 °C for 2 h [35]. The solution started to form silver nanoparticles as it changed color from yellow to brown. To further characterize the AgNPs-S, they were centrifuged, washed with ethanol, dried, and subsequently utilized for various biological assays.

### 2.8. Characterization of AgNPs-S

The first step in characterizing the AgNPs-S was the spectrophotometric analysis. A range of 300 to 700 nm was scanned with a resolution of 1 nm for AgNPs-S [36]. Fourier transform infrared spectroscopy (FT-IR) was further used to investigate the potential interaction between saponins and AgNO_3_ (Bruker^®^, Billerica, MA, USA). The spectra were recorded in the range of 500 to 4000 cm^−1^ with 32 scans and a resolution of 4 cm^−1^ [37]. To estimate the shape and size of AgNPs-S, TEM measurements were also performed. The TEM analysis was carried out using a JEM-1400 Plus, Jeol, India. A TEM analysis was performed after smearing the AgNPs-S sample in a grid of carbon-coated copper and allowing it to evaporate for 1 h under a vacuum dryer [38].

### 2.9. Antibacterial Activity of AgNPs-S

An agar well diffusion technique was used to test the antibacterial activity of AgNPs-S against various Gram-positive and Gram-negative pathogenic bacteria, such as *Chromobacterium violaceum* MTCC-2656 (*C. violaceum*), *Pseudomonas aeruginosa* MTCC-741 (*P. aeruginosa*), *Escherichia coli* MTCC-9537 (*E. coli*), *Bacillus subtilis* MTCC-121 (*B. subtilis*), *Proteus vulgaris* MTCC-426 (*P. vulgaris*), *Serratia marcescens* MTCC-97 (*S. marcescens*), *Enterococcus faecalis* MTCC-439 (*E. faecalis*), and *Staphylococcus aureus* MTCC-96 (*S. aureus*) [39]. Using a sterile swab, the cultures of each bacterial strain, which had been developed overnight, were streaked onto an MHB agar plate. In the following step, using a sterile cork borer, wells were punctured, and AgNPs-S were transferred into each well. The plates were incubated at 37 °C for 24 h. Furthermore, the incubated samples were determined by measuring the inhibition zone.

### 2.10. Determination of Minimum Inhibitory Concentration (MIC)

The standard broth dilution assay was used to evaluate the MIC values of AgNPs-S against different bacterial pathogens [40]. The AgNPs-S were used in a series of double dilutions in MHB with an active bacterial culture (108 CFU/mL, 0.5 McFarland standard) to determine the MIC. The concentrations of the sample ranged from 1698.7 µg/mL to 1.65 µg/mL. A control was prepared using only inoculated broth, which was incubated at 37 °C for 24 h. The smallest concentration of AgNPs-S that prevents any growth on the tubes is known as the MIC. To verify the MIC value, the turbidity of the tubes was measured before and after incubation.

### 2.11. Determination of Nucleic Acid Leakage

Cell membrane integrity can be observed via the liberation of the cytoplasmic components of a cell that are indicative of the integrity of its membrane [41]. The incubation of *P. aeruginosa* bacteria was achieved using an LB medium at 37 °C for 12 h. Except for the control, the log-phase culture was treated with AgNPs-S (1 × MIC, 2 × MIC). After that, the incubation of samples was carried out at 37 °C for 6 and 24 h. The samples were filtered as soon as they were collected using an organic membrane with a 0.2 μm filter. The optical density of the supernatant was measured at 260 nm to estimate the amount of DNA and RNA released from the cytoplasm.

### 2.12. Determination of Protein Leakage

The effect of AgNPs-S on cell integrity was further determined by checking the release of proteins after the treatment of the AgNPs-S. Bradford’s method was used to determine the protein concentrations in the supernatants [42]. The log-phase cultures of *P. aeruginosa* were treated with AgNPs-S (1 × MIC, 2 × MIC), except the control, and incubated at 37 °C for 6 h and 24 h. Centrifugation at 6000 rpm for 10 min at 4 °C was performed after incubation. The protein concentration was determined by adding 200 µL of the supernatant to 800 µL of the Bradford reagent at 595 nm via a UV spectrophotometer (UV-2600, Shimadzu, Japan). Bovine serum albumin (BSA) was used as a standard protein.

### 2.13. Determination of Antibiofilm Activity

Test tubes made of glass were used to investigate the antibiofilm effects of AgNPs-S as a hydrophilic surface [43]. Briefly, tubes containing 1 mL of an active bacterial culture and 500 µL of AgNPs-S (sub-MICs) received 3 mL of sterilized LB medium. After thorough mixing, the tubes were incubated in a shaker for 72 h at room temperature. After the tubes had been incubated, planktonic cells were taken out and washed with PBS. Inside the tubes, the biofilm that had grown was stained with crystal violet. By washing the stained biofilm with PBS and eliminating the excess dye, the developed biofilm was dissolved in acetic acid and its absorbance was measured at 595 nm using the UV spectrophotometer (UV-2600, Shimadzu, Japan). Controls for the growth of biofilms were made using an LB medium containing individual bacterial strains. Biofilm inhibition was estimated as follows:

O.D._control_ − O.D._test_/O.D._control_ × 100

### 2.14. Determination of the Anti-QS Activity of AgNPs-S

The AgNPs-S were evaluated for their anti-QS properties against *C. violaceum*, *P. aeruginosa*, and *S. marcescens* via a well-diffusion assay. Bacterial cultures grown overnight were spread over Petri plates, and a gel puncture was used to make wells. The wells were punctured, 50 µL of AgNPs-S was added, and the plates were then incubated at 37 °C for 24 h. The anti-QS effects were seen the next day as a zone of clearance [44].

### 2.15. Assessment of Violacein Pigment Production in C. violaceum

In order to quantify violacein production, the standard procedure was followed [45]. A culture of *C. violaceum* was grown for 18 h at 30 °C without and with sub-MIC concentrations of AgNPs-S. Violacein pigment and bacterial cells were separated using centrifugation at 10,000 rpm for five minutes. To dissolve the pigment, the pellet was violently vortexed in 1 mL of DMSO for 5 min. The suspension was centrifuged again to spin down the bacterial debris. For the measurement of supernatant absorbance, a UV spectrophotometer was used (UV-2600, Shimadzu, Japan). The inhibition of violacein pigment production was estimated as follows:

O.D._control_ − O.D._test_/O.D._control_ × 100

### 2.16. Assessment of Pyocyanin Pigment Production in P. aeruginosa

Following the established standard protocols, the impact of AgNPs-S on the synthesis of pyocyanin in *P. aeruginosa* was examined in both the presence and the absence of AgNPs-S. [46]. The growth of *P. aeruginosa* in the LB medium in the presence and absence of sub-MICs of AgNPs-S took place overnight at 37 °C. Afterwards, 5 mL of the grown culture was centrifuged at 10,000 rpm for 10 min to collect the supernatant. The pyocyanin pigment production was then extracted from the supernatant of culture via extraction with 3 mL of chloroform. In the following step, the organic phase was collected and re-extracted with 1.2 mL of 0.2 N HCl. As a final step, the aqueous phase was taken, and absorbance was determined using a UV spectrophotometer (UV-2600, Shimadzu, Japan). The inhibition of pyocyanin pigment production was estimated as described above.

### 2.17. Assessment of Prodigiosin Pigment Production in S. marcescens

The effect of AgNPs-S on the production of prodigiosin synthesis in *S. marcescens* was investigated in both the presence and the absence of AgNPs-S by following standard procedures [47]. A test culture of *S. marcescens* was grown overnight at 30 °C in a sterile LB medium with and without AgNPs-S. After incubation, bacterial cells were collected using centrifugation for 10 min at 10,000 rpm. The resulting cell pellet was thoroughly stirred at room temperature into acidified ethanol (96 mL ethanol + 4 mL 1 M HCl). To eliminate cell debris, the mixture was centrifuged once more. The absorbance of the supernatant at 534 nm was then calculated using a spectrophotometer (UV-2600, Shimadzu, Japan). The inhibition of prodigiosin pigment production was estimated as described above.

### 2.18. Determination of DPPH Free-Radical-Scavenging Activity

The antioxidant activity of AgNPs-S was assessed against DPPH free radicals [48]. A freshly prepared DPPH solution in methanol (1 mM) was combined with equal volumes of different concentrations of AgNPs-S (1–1000 µg/mL) and mixed thoroughly. Following that, the solution was incubated for 30 min at room temperature in the dark. After incubation, a UV spectrophotometer (UV-2600, Shimadzu, Japan) was used to measure absorbance at 517 nm. Methanol served as a blank, and DPPH served as the control. The percentage of inhibition was used to calculate the free-radical-scavenging activity according to the following formula:

% Scavenging activity = Pc − Ps/Pc × 100

where Pc is the absorbance of the control and Ps is the absorption of the AgNPs-S.

### 2.19. Determination of the Cytotoxic Potential of AgNPs-S

A549 (human non-small-cell lung cancer) and HEK293 (human embryonic kidney cells) were used to investigate the cytotoxic activity of the biosynthesized AgNps-S. Cells were raised in Dulbecco’s Modified Eagle’s Medium (DMEM) (MP Biomedicals, Eschwege, Germany) with 10,000 units/mL penicillin, 5 milligrams/mL streptomycin antibiotic solution, and 10% fetal bovine serum (Hi-Media, Mumbai, India) in T-25 flasks (25 cm^2^) at 37 °C in humidified atmospheres with 5% CO_2_. Cells were seeded in 96-well plates at a density of 10^4^ cells per well after achieving 80% confluency. A hemocytometer was used to determine the viability of the cells after staining with Trypan Blue (0.4%) (Hi-Media^®^, Mumbai, India). In the following step, the cells were treated with different concentrations of AgNPs-S (1–1000 µg/mL) for 48 h. Following the removal of the plate from the incubator, the AgNPs-S-containing medium was aspirated. Each well was then incubated at 37 °C for 3 h under a humidified atmosphere (5% CO_2_) with 200 µL of the medium containing 10% MTT reagent (MP Biomedicals, Eschwege, Germany). The formazan crystals were dissolved in 100 L of DMSO (Merck, Darmstadt, Germany) after the medium had been removed. With the help of an ELISA reader (EL10A, Biobase, China), the absorbance at 570 and 630 nm was measured in order to calculate the concentration of formazan crystals in the sample. To calculate the percentage of growth inhibition, a calculation was carried out by subtracting the values of the background and blank. Cisplatin was used as a positive control in this assay.

### 2.20. Statistical Analysis

Results are presented as the mean ± SD of the number of experiments performed. The significance of the results was determined for the treatments using an ordinary one-way ANOVA followed by Bonferroni’s multiple comparisons test at *p* < 0.05. The analyses were carried out using the Graph Pad Prism software 8.0.

## 3. Results

### 3.1. Extraction and Confirmation of Saponins

**Figure 1:**
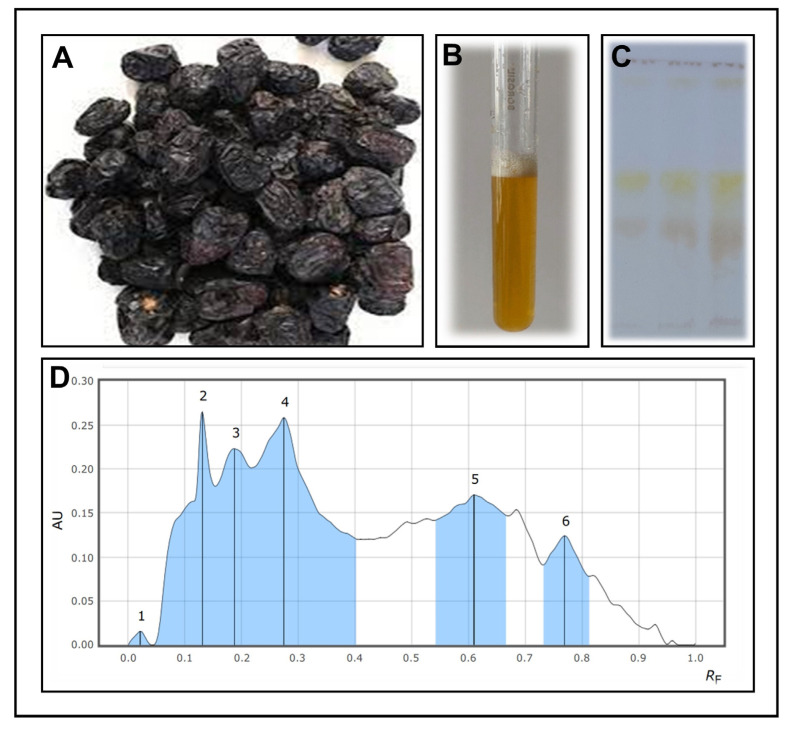
(**A**) Ajwa date fruits, (**B**) foam test of the saponin-enriched fractions of the Ajwa dates, (**C**) an HPTLC image of Ajwa dates with saponin-enriched fractions, (**D**) analyses of saponin-enriched fractions of Ajwa dates showing the separation of different compounds using HPTLC.

The solvent-extraction method was used to extract crude saponins from the Ajwa dates. An initial qualitative test for saponins was conducted using the froth test, which was found to be successful in demonstrating the presence of saponins. In the HPTLC analysis, different compounds were detected with retention factors (Rf) of 0.021, 0.131, 0.187, 0.274, 0.610, and 0.769 for the extracted saponins from the extraction process (Figure 1A–D). Based on the spectrophotometric analysis, it was confirmed that the total crude saponin-enriched fraction yield was 415.89 µg/mL of crude saponins.

### 3.2. Synthesis and Characterization of AgNPs-S

**Figure 2:**
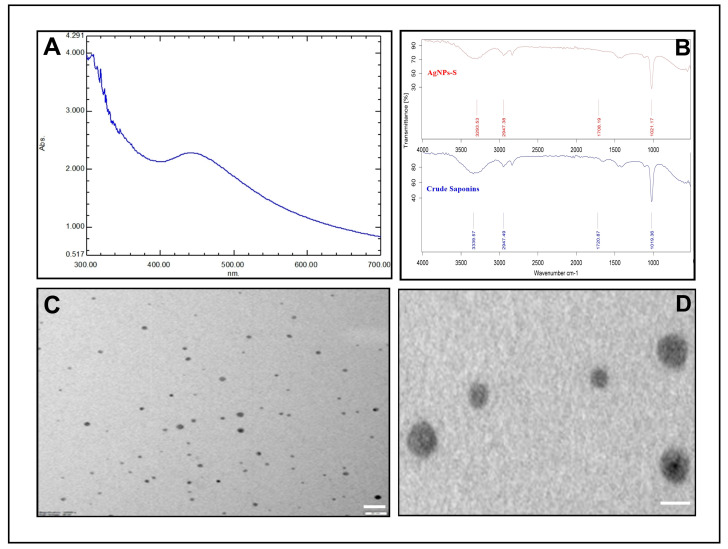
Characterization of AgNPs-S. (**A**) UV–visible absorption spectra of AgNPs-S. (**B**) FT-IR analysis of AgNPs-S. (**C**,**D**) Morphological analysis of AgNPs-S via TEM analysis, scale bar = 20 nm. (**D**) Enlargement of AgNPs-S, scale bar = 10 nm.

AgNPs-S biosynthesis was achieved after extracting saponins from Ajwa dates and mixing them with a silver nitrate solution. During the incubation period, the sample color changed from yellow to dark brown. UV–visible spectroscopy is an important tool that is employed to detect AgNPs. UV–Vis spectroscopy was used as the primary method of checking the formation and constancy of synthesized AgNPs-S. A spectroscopy measurement after 24 h expressed an absorption spectrum with a peak maximum of 440 nm for the synthesized AgNPs-S (Figure 2A). Additionally, spectroscopy measurements were performed on the synthesized AgNPs-S after a period of one week, and the spectroscopic results showed no noticeable variation in the spectra of the nanoparticles, providing further evidence of their stability.

The FTIR spectra of crude saponins and AgNPs-S are shown in Figure 2. The FTIR spectra of AgNPs-S showed absorption bands at 1021.17 cm^−1^, 1708.19 cm^−1^, 2947.38 cm^−1^, and 3293.53 cm^−1^, corresponding to C–O stretching vibration of ethers or glycosides, C=O stretching vibration of carbonyls, C–H stretching vibration of alkanes, and O–H stretching vibration of hydroxyl groups. The FTIR spectra of crude saponins showed intense bands at 1019.36 cm^−1^, 1720.87 cm^−1^, 2947.49 cm^−1^, and 3339.57 cm^−1^, and a slight shift in the peak positions of IR bands in crude saponins and AgNPs-S was observed that indicates the functional groups and interactions involved in the synthesis and capping process. The size and morphology of the AgNPs-S were examined using TEM analysis. The resultant TEM images obtained at 120,000× magnifications and 80 KV are shown in Figure 2C,D. The TEM analysis revealed that the particles are spherical in nature and polydisperse. The sizes of the AgNPs-S, calculated via TEM analysis, were 2–10 nm.

### 3.3. Antibacterial Potential of AgNPs-S

**Figure 3:**
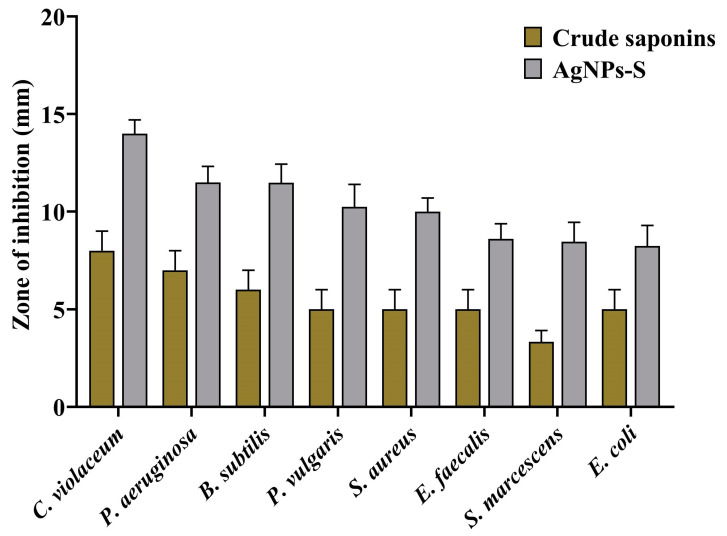
Antibacterial activity of crude saponins and AgNPs-S against different Gram-positive and Gram-negative bacterial pathogens. Values are represented as the mean ± SD of three independent experiments.

**Figure 4:**
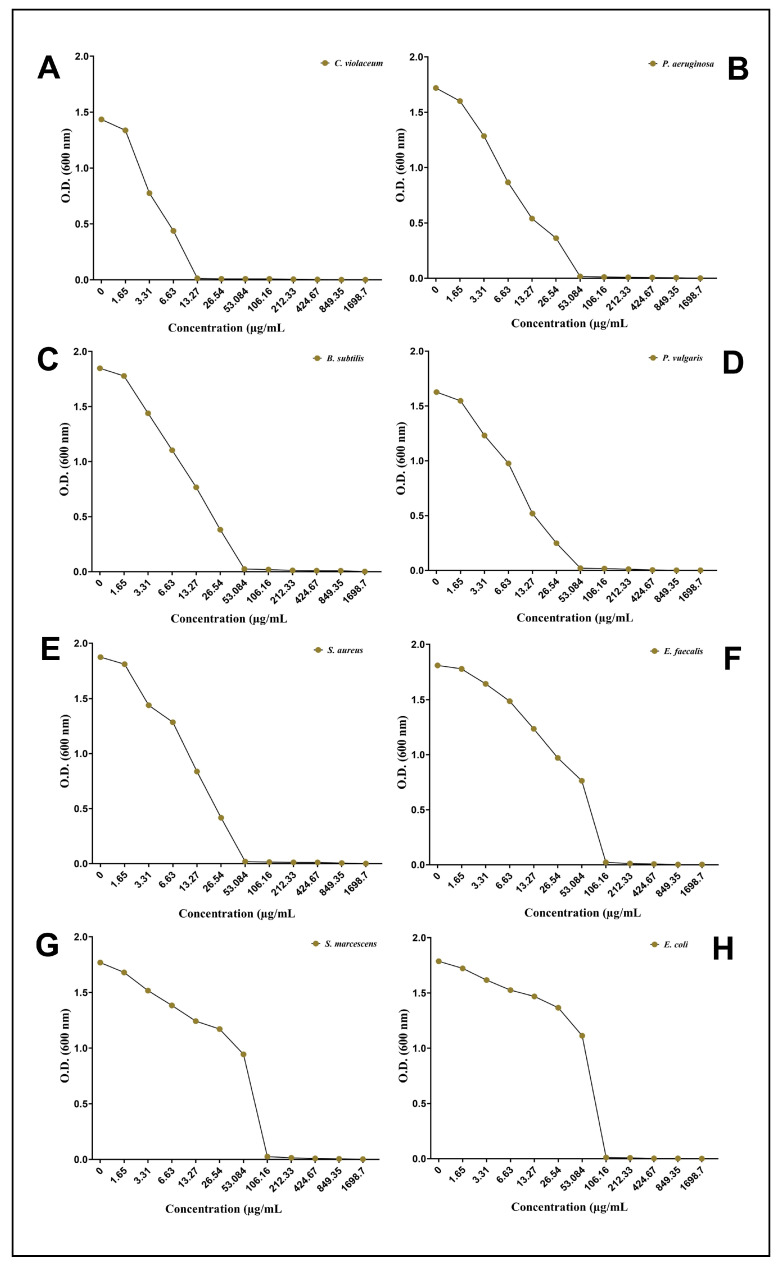
Determination of MIC after taking the optical density at 600 nm: (**A**) *C. violaceum*, (**B**) *P. aeruginosa*, (**C**) *B. subtilis*, (**D**) *P. vulgaris*, (**E**) *S. aureus*, (**F**) *E. faecalis*, (**G**) *S. marcescens*, and (**H**) *E. coli*.

A well diffusion assay demonstrating the antibacterial properties of the biosynthesized AgNPs-S against Gram-positive and Gram-negative bacteria shows that they possess antibacterial properties. Zones of inhibition were used as a measure of antibacterial activity when assessing the results. The zone of inhibition was 14.00 mm against *C. violaceum*, 11.50 mm against *P. aeruginosa*, 11.48 mm against *B. subtilis*, 10.24 mm against *P. vulgaris*, 10.00 mm against *S. aureus*, 8.60 mm against *E. faecalis*, 8.46 mm against *S. marcescens*, and 8.24 mm against *E. coli* (Figure 3). A broth microdilution method was used to estimate the MIC of AgNPs-S against the selected pathogens. Furthermore, in order to determine the MIC of AgNPs-S against the tested pathogens, a broth microdilution method was used. The AgNPs-S had MIC values of 13.27 µg/mL for *C. violaceum*, 53.08 µg/mL for *P. aeruginosa*, *B. subtilis*, *P. vulgaris*, and *S. aureus*, and 106.16 µg/mL for *E. faecalis*, *S. marcescens*, and *E. coli* (Figure 4).

### 3.4. Determination of the Effect of AgNPs-S on Nucleic Acids and Protein Leakage

**Figure 5:**
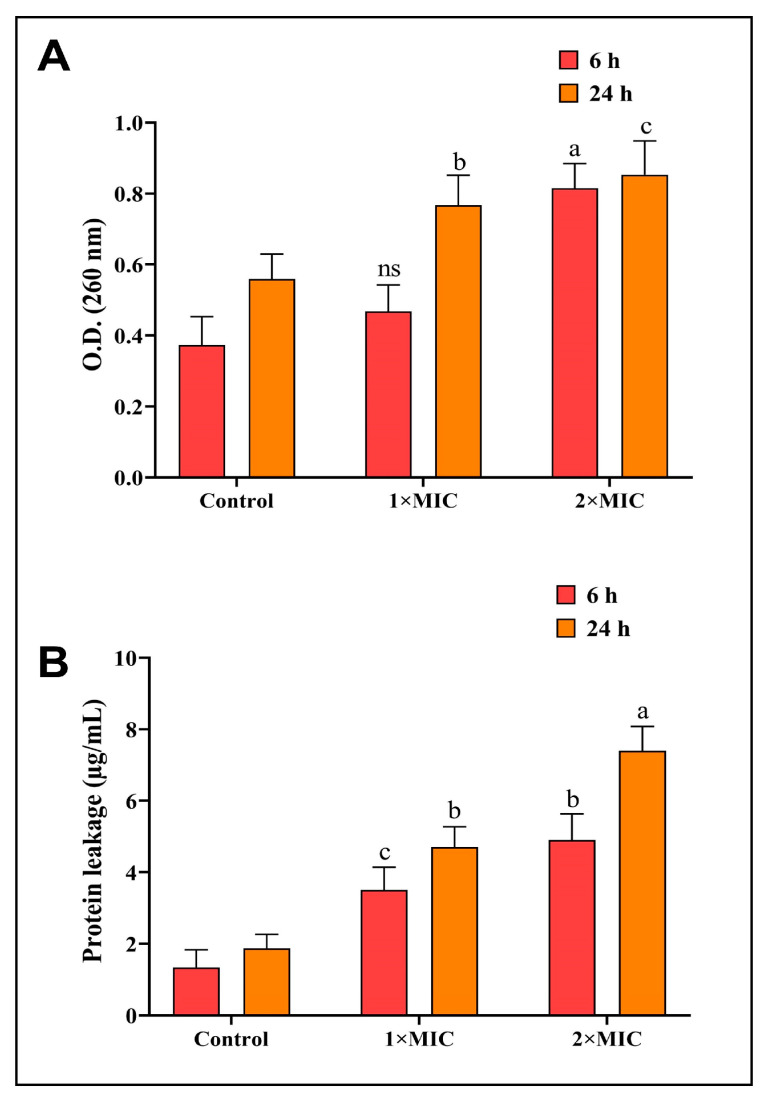
(**A**) AgNPs-S led to DNA and RNA leakage from *P. aeruginosa* bacteria. (**B**) AgNPs-S led to essential protein leakage from *P. aeruginosa* bacteria. Values are represented as the mean ± SD of three independent experiments. Different superscript letters indicate significant differences at *p* ≤ 0.05 with respect to the control.

As a possible mechanism of antibacterial action, the AgNPs-S were tested in order to determine whether they could suppress *P. aeruginosa* by leaking DNA and RNA via the membranes of the bacteria. When AgNPs-S were added to the bacterial culture, the results showed that the nucleic acids of the bacterial cell content were released into the medium, and these nucleic acid contents were released at an increasing rate with increasing doses of AgNPs-S. As a result of the *P. aeruginosa* treatment with supernatants of AgNPs-S (1 × MIC, 2 × MIC) for 6 and 24 h, the optical density (OD260) of the tested aliquot was much higher than that of the untreated controls. It is also worth noting that the optical density readings were observed to increase following the incubation intervals for 6 and 24 h (Figure 5A). There is an indication that AgNPs-S caused damage to the cell membrane of *P. aeruginosa*, which led to the disruption to the cell membrane and caused a leaking of macromolecules, including DNA and RNA.

A further effect of AgNPs-S was observed via the disruption of proteins in the cell membranes of bacterial cells. According to the results shown in Figure 5B, AgNPs-S caused damage to the bacterial cell membrane of *P. aeruginosa*, which caused essential proteins to leak into the environment. In the untreated control, the leakage of proteins at the start of the experiment was much lower than the drip of proteins in the AgNPs-S-treated group. There was an increase in protein leakage not only with an increase in AgNPs-S concentrations, but also with an upsurge in the duration of the treatment. AgNPs-S caused a reduction in the content of cellular proteins due to their ability to penetrate and disrupt the membranes of cells.

### 3.5. Antibiofilm Potential of AgNPs-S

**Figure 6:**
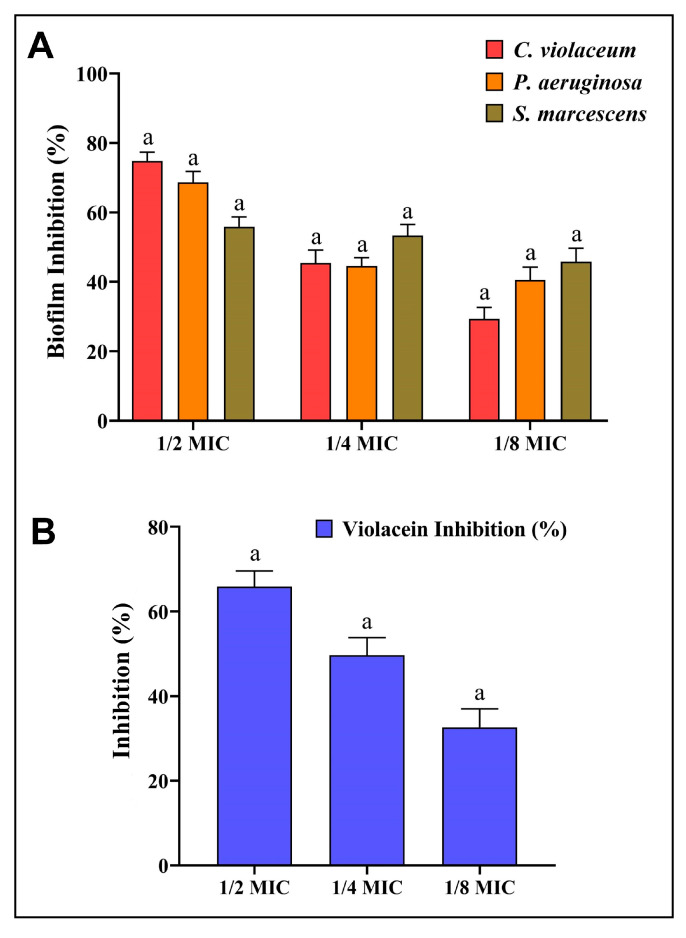
(**A**) Analysis of the quantitative inhibition of biofilm production using AgNPs-S. (**B**). Analysis of the quantitative inhibition of violacein in *C. violaceum* using AgNPs-S. Values are presented as the mean ± SD of three independent experiments. Different superscript letters indicate significant differences at *p* ≤ 0.05 with respect to the control.

In the presence of AgNPs-S, *C. violaceum*, *P. aeruginosa*, and *S. marcescens* were observed to produce less biofilm in a concentration-dependent manner. At concentrations below the MIC, AgNPs-S reduced biofilm formation by 74.79%, 45.40%, and 29.32% in *C. violaceum*; 68.63%, 44.52%, and 40.55% in *P. aeruginosa*, and 55.87%, 53.34%; and 45.81% in *S. marcescens* (Figure 6A).

### 3.6. Anti-QS Potential of AgNPs-S

AgNPs-S were initially examined for their impact on *C. violaceum* QS activity. AgNPs-S prevented *C. violaceum* from producing violacein pigment at sub-MIC concentrations, which would indicate an inhibition of QS activity. Violacein production decreased by 65.89%, 49.69%, and 32.63% after treatment with 1/2, 1/4, and 1/8 MIC, respectively (Figure 6B). Furthermore, no inhibition was observed for the growth of *C. violaceum* when it was given treatments with AgNPs-S at sub-MIC levels.

**Figure 7:**
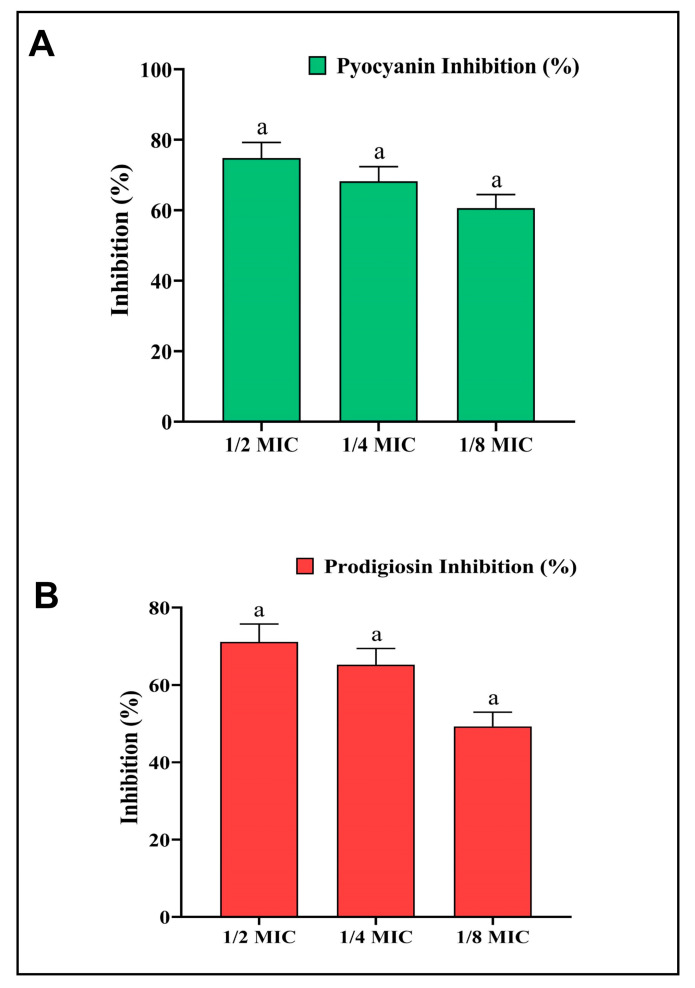
(**A**) Analysis of the quantitative inhibition of pyocyanin production in *P. aeruginosa* using AgNPs-S. (**B**) Analysis of the quantitative inhibition of prodigiosin in *S. marcescens* using AgNPs-S. Values are presented as the mean ± SD of three independent experiments. Different superscript letters indicate significant differences at *p* ≤ 0.05 with respect to the control.

The AgNPs-S were further studied for the inhibition of pyocyanin pigment to find out whether they could be effective in inhibiting the QS system of *P. aeruginosa*. Furthermore, pyocyanin is considered a powerful virulent factor that contributes to *P. aeruginosa* pathogenesis, virulence, and growth. A concentration-dependent reduction in pyocyanin formation at sub-MIC concentrations was achieved with AgNPs-S, and this reduction was measured at 74.83%, 68.21%, and 60.59%, respectively (Figure 7A).

Moreover, the anti-QS activity of AgNPs-S was also studied in relation to the inhibition of prodigiosin activity in *S. marcescens*. Bacteria produce a pigment called prodigiosin, which is believed to play an essential role in promoting the growth, pathogenesis, and virulence of the organism. Prodigiosin production was reduced at sub-MIC doses by 71.10%, 65.22%, and 49.28% in a dose-dependent manner (Figure 7B).

### 3.7. In Vitro Antioxidant and Cytotoxic Potential of AgNPs-S

**Figure 8:**
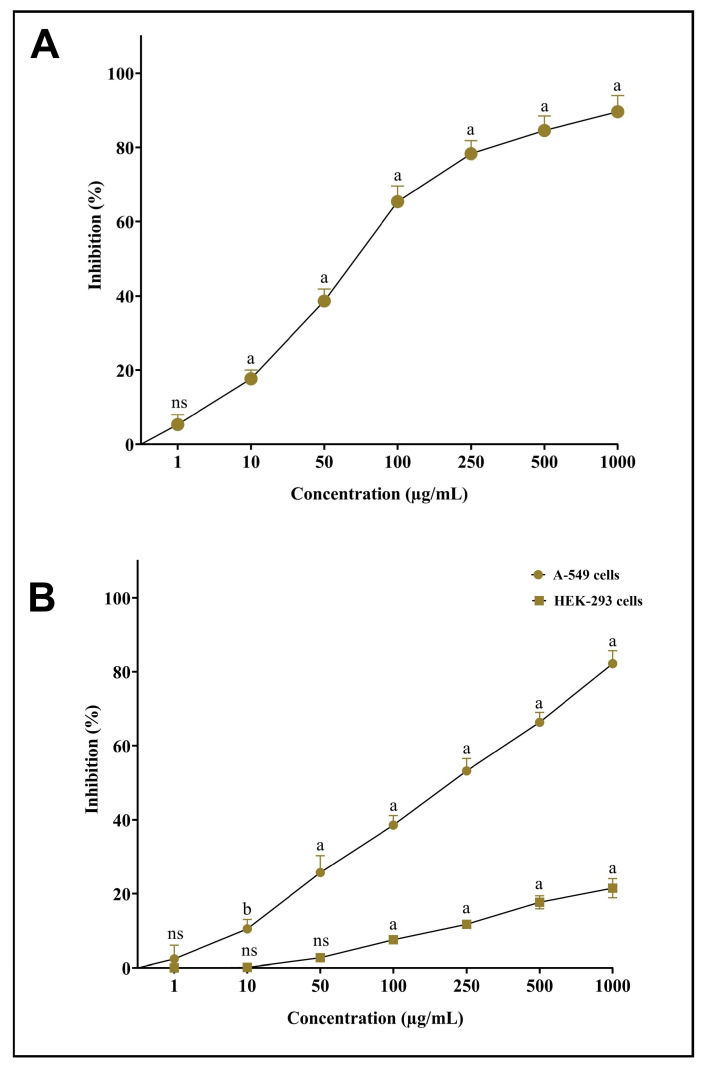
(**A**) Antioxidant potential of AgNPs-S against DPPH free radicals. (**B**) Cytotoxic activity of AgNPs-S against A549 human non-small-cell lung-cancer cells and normal human embryonic kidney cells (HEK-293). Values are presented as the mean ± SD of three independent experiments. Different superscript letters indicate significant differences at *p* ≤ 0.05 with respect to the control.

As part of the assessment of newly synthesized AgNPs-S and their antioxidant potential, they was tested against DPPH free radicals. Our novel synthesized AgNPs-S was observed to have good radical-scavenging abilities when exposed to DPPH free radicals. The antioxidant action of AgNPs-S was obtained in a dose-dependent manner, meaning that the antioxidant potency tended to rise with the increase in the sample concentration (Figure 8A). An MTT assay was used to assess the potential cytotoxic properties of AgNPs-S in contrast to non-small-cell lung cancer cells (A549) and normal human embryonic kidney cells (HEK-293). The obtained results indicated that the A549 cancer cells were inhibited in a dose-dependent manner after the treatment with AgNPs-S, whereas a low cytotoxicity of AgNPs-S was observed against HEK-293 cells at higher concentrations (Figure 8B).

## 4. Discussion

Natural compounds known as saponins can be found in a variety of plants, especially in legume seeds and roots, herbs, vegetables, and fruits [49]. Their name is derived from their ability to form soapy lathers when mixed with water. An important characteristic of saponins is that they are glycosides, meaning that they are a mixture of sugar molecules and non-sugar molecules. The non-sugar molecule in saponins is usually a steroid or triterpenoid, while saponins contain a variety of sugar molecules that give rise to a range of biological activities [50]. Saponins are known for forming stable foams or froths when they are shaken with water, one of their most well-known properties. Due to their natural foaming properties, they are often used in applications such as detergents, shampoos, and soaps, where they can act as natural foaming agents [51]. As well as being capable of providing surfactant properties, saponins have also been shown to possess a variety of biological properties that include antimicrobial, antifungal, anti-inflammatory, and anticancer properties [52,53,54,55].

In this study, we investigated the antibiofilm, antibacterial, and anti-QS activities of novel AgNPs synthesized from saponins derived from Ajwa dates. Recently, interest in the potential effectiveness of AgNPs as antibacterial agents has experienced an uptick due to their distinctive properties, including their improved surface area, improved reactivity, and potential to overcome antibiotic resistance [11,12,13]. Ajwa dates, known for their rich saponin content, proved to be an intriguing natural source for the development of AgNPs by means of potential antimicrobial activity [56]. As a result of the characterization studies, it was confirmed that AgNPs can be successfully synthesized using the saponins derived from Ajwa dates. The UV–Vis spectroscopy results revealed characteristic absorption peaks in the range associated with the formation of AgNPs, indicating that nanoparticles were formed during the synthesis process. The Fourier-transform infrared spectroscopy (FTIR) investigation revealed various functional groups from Ajwa date saponins that contribute to the stability and capping of AgNPs. The C-H stretching vibration of alkanes is usually seen around 2900 cm^−1^ and indicates the presence of aliphatic hydrocarbon chains in saponins. This peak may not change significantly after the synthesis of AgNPs, as the hydrocarbon chains are not involved in the reduction or capping process [57]. The C=O stretching vibration of carbonyls is usually seen around 1700 cm^−1^ and indicates the presence of carbonyl groups (C=O) in saponins. These groups can be part of ketones, esters, or carboxylic acids. This peak may shift or change in intensity after the synthesis of AgNPs, as the carbonyl groups can be involved in the reduction or capping process. For example, carboxylic acids can donate electrons to silver ions and form carboxylate groups that bind to the surface of AgNPs [58]. The O-H stretching vibration of alcohols or phenols is usually seen around 3400 cm^−1^ and indicates the presence of hydroxyl groups (O-H) in saponins. These groups can be part of sugars, glycosides, or phenolic compounds. This peak may shift or change in intensity after the synthesis of AgNPs, as the hydroxyl groups can be involved in the reduction or capping process. For example, phenols can donate electrons to silver ions and form phenolate groups that bind to the surface of AgNPs [59]. The C-O stretching vibration is found in ethers and glycosides. This peak is usually seen around 1100 cm^−1^ and indicates the presence of ether or glycosidic linkages (C-O-C) in saponins. These linkages connect different sugar units or aglycones in saponins. This peak may not change significantly after the synthesis of AgNPs, as the ether or glycosidic linkages are not involved in the reduction or capping process [59]. In the present study, a similar pattern of FTIR spectra was observed for crude saponins and AgNPs-S, which confirms the synthesis and capping process of AgNPs using crude saponins. Further confirmation of the successful synthesis of AgNPs was provided by transmission electron microscopy (TEM) images that showed polydispersed AgNPs 2–10 nm in size and with spherical shapes. As reported earlier [57], saponin-capped silver nanotriangles were prepared in an aqueous system using a *Trigonella foenum-graecum* seed extract. The synthesized nanoparticles were crystalline and triangular in shape, with an edge length of approximately 80 nm. Using UV/Vis spectroscopy, it was observed that the synthesized saponin-capped silver nanotriangles showed three absorption peaks at 360 nm, 432 nm, and 702 nm. For more than six months, these peaks remained in almost the same position, which confirms that the silver nanotriangles have a high level of stability. The FTIR spectra confirmed the presence of saponin on the surface of the silver nanotriangles, which acted as a capping agent and prevented their aggregation. In one study, AgNPs were synthesized using saponin-rich/poor leaf extracts from *Ocimum tenuiflorum* and *Phyllanthus urinaria* [60]. The results of the study showed that leaf extracts rich in saponins (WE) produced AgNPs that were smaller, more uniform in size, and more stable than extracts that were low in saponins (EE). The AgNPs synthesized from WE had an average size of 9.6 nm, and an average size of 18.8 nm was measured for those synthesized from EE. There was a zeta potential of −29.3 mV for the AgNPs synthesized from WE and −17.4 mV for the AgNPs synthesized from EE. As a result of the FTIR analysis, it was confirmed that saponin was present on the surface of the AgNPs synthesized from WE as a capping agent. It was evident from the TEM images that the AgNPs synthesized from WE exhibited a spherical shape, while those synthesized from EE displayed an irregular shape. Similarly, AgNPs were also synthesized and characterized by [61] using saponin extracts from *Simarouba glauca* oil seed meal. With an average size of 10 nm, the synthesized AgNPs were spherical in shape. There were intense bands in the FTIR spectrum of saponin at 3350 cm^−1^, 2827 cm^−1^, 1704 cm^−1^, and 1024 cm^−1^, whereas the AgNPs showed absorption bands at 3375 cm^−1^, 2928 cm^−1^, 1707 cm^−1^, and 1024 cm^−1^, corresponding to O–H stretching vibrations, C–H stretching vibrations, and symmetric and asymmetric C–O–C stretching vibrations of carboxyl groups, respectively. In saponin and the synthesized AgNPs, there was a slight shift in the peak positions of the IR bands caused by the reduction process. The results of this study confirm that saponin can adsorb on the surfaces of AgNPs and that saponins can cap and stabilize nanoparticles.

In recent years, there has been rising concern about the formation of biofilms and the emergence of antibiotic resistance within various domains, such as pharmaceutical medicine, agriculture, and other industries [62]. A biofilm is composed of a variety of microbes, such as bacteria, fungi, and algae, which attach to surfaces and form an extracellular polymeric substance (EPS). Additionally, various reports suggest that biofilms could play a significant role in the development of numerous infections. Biofilm formation can occur on medical apparatuses, implants, and natural surfaces [63,64,65]. In the presence of a biofilm, bacteria exhibited significant levels of resistance to antibiotics, as well as being immune to the immune response of the host. [66]. A number of factors cause this resistance, including physical barriers, altered phenotypes, quorum sensing, and persistent cells [67]. In the healthcare setting, biofilms and antibiotic resistance pose significant challenges that need to be addressed. Infections associated with biofilms are often chronic and hard to treat and can result in the recurrence of infections even after the initial treatment has been completed. A second factor contributing to the spread of antibiotic resistance within a microbiome is the horizontal gene transfer of antibiotic resistance genes between bacteria and within their biofilm [68,69].

It is essential to take a multifaceted approach to addressing the issue of biofilms and antibiotic resistance in order to resolve this issue. A number of strategies are being explored, including the formulation of novel antimicrobial compounds that are specifically targeted in biofilms, the design of surface coatings that prevent biofilm formation, the use of combination therapies to treat multiple pathways, and the exploration of alternative treatments such as bacteriophages [70,71,72,73,74]. During the last few years, a tremendous amount of consideration has been paid to the inhibition of biofilm formation through targeting the QS mechanism. During the QS process, bacteria communicate with one another and coordinate their activities within a biofilm [75]. By interfering with the process of QS, the communication between bacterial cells is disrupted, causing the breakdown of the biofilm and the associated resistance mechanisms. As a consequence of this disruption, mature biofilms cannot be formed, resulting in the bacteria being more susceptible to antibiotics and other treatments [76]. Therefore, targeting the QS mechanism presents a promising avenue for inhibiting biofilm formation, reducing antibiotic resistance, and improving the effectiveness of antimicrobial treatment.

Saponins have long been recognized for their antimicrobial properties, which can be attributed to their amphiphilic nature and ability to disrupt bacterial cell membranes. The hydrophilic glycone portion and the lipophilic aglycone moiety of saponins contribute to their interactions with bacterial membranes. Saponins are inserted into the lipid bilayer, causing disruptions in the membrane’s integrity. This disruption leads to increased permeability, the leakage of cellular contents, and eventual bacterial cell death. Moreover, saponins can also interfere with bacterial quorum sensing, a process critical for bacterial communication and virulence [77]. The combination of saponins with AgNPs has garnered interest as a means of enhancing antibacterial efficacy. Saponins can serve as stabilizing agents for AgNPs, preventing aggregation and maintaining their colloidal stability. This synergistic approach leverages the inherent antibacterial mechanisms of both compounds, leading to a cumulative effect on bacterial inhibition [78]. The newly synthesized AgNPs-S and their antibacterial activity were assessed against a variety of bacterial strains in order to determine their effectiveness. A pronounced inhibitory effect was observed in the zone of inhibition assays, indicating that AgNP-S can be effective in the fight against bacterial growth. Furthermore, the determination of the MIC values indicated that AgNPs-S can be effective against bacteria at low concentrations. In view of these findings, it is clear that AgNPs derived from the saponins of Ajwa dates possess potent antibacterial properties. However, the MIC values of AgNPs were found to be different against different Gram-positive and Gram-negative bacterial pathogens. This might be for several reasons; for instance, Gram-positive bacteria have a relatively thick peptidoglycan layer in their cell walls, which provides structural integrity and plays a crucial role in resisting the entry of foreign substances, including antibacterial agents [79]. This thicker peptidoglycan layer can act as a barrier, making it more difficult for antibacterial agents to penetrate and reach their target sites within the bacterial cell. As a result, Gram-positive bacteria may require higher concentrations of an antibacterial agent to achieve the same inhibitory effect, leading to a higher MIC value [80]. On the other hand, Gram-negative bacteria have an additional outer membrane composed of lipopolysaccharides (LPS) that surrounds their thin peptidoglycan layer. This outer membrane serves as an extra barrier and can restrict the entry of hydrophobic molecules, including some antibiotics, into the bacterial cell [81]. Therefore, Gram-negative bacteria might exhibit stronger resistance to certain antibacterial agents, resulting in higher MIC values. Moreover, Gram-negative bacteria possess efflux pumps that actively pump out various compounds, including antibiotics, from the bacterial cell. This efflux mechanism contributes to antibiotic resistance by quickly expelling the drug and reducing its effective concentration within the cell, which can lead to elevated MIC values. Therefore, the differences in cell wall structures, membrane properties, and resistance mechanisms between Gram-positive and Gram-negative bacteria can all contribute to variations in MIC values for antibacterial agents targeting these different types of bacteria [82].

AgNPs are well known to exert antibacterial effects against a variety of bacteria, including multi-drug-resistant strains. There are several ways in which AgNPs can interact with bacteria, depending on their size, shape, surface charge, and coating. Upon attachment to the bacterial cell wall, AgNPs can disrupt its integrity and leak cytoplasmic content and membrane potential [83,84]. DNA and RNA can be damaged by AgNPs that penetrate into the bacterial cytoplasm and bind to nucleic acids, preventing their replication, transcription, and translation [84]. Furthermore, AgNPs can also produce reactive oxygen species (ROS) and free radicals, which are capable of inducing DNA damage [83]. Bacterial proteins and enzymes can be denatured, aggregated, or inhibited by AgNPs [83,84]. Additionally, AgNPs can impact ribosomes and tRNAs, which are essential for protein synthesis [84]. The oxidative phosphorylation and ATP synthesis of bacteria can be interfered with by AgNPs [83,84]. It is possible for these mechanisms to lead to the death of bacteria through apoptosis or necrosis [83,84]. In the present study, synthesized AgNPs-S were able to leak nucleic acids and protein at concentrations of 1 × MIC and 2 × MIC after 6 h and 24 h in *P. aeruginosa*.

Furthermore, AgNPs-S also displayed remarkable antibiofilm activity. As AgNPs-S are capable of inhibiting biofilm formation, it indicates that they can be used for the prevention of bacterial infections and to fight against antibiotic resistance in the future. The disruption of biofilms by AgNPs-S indicates their potential to augment the effectiveness of conventional antibiotics to a considerable extent. This study shows that AgNPs-S have significant antibiofilm potential, which is consistent with the results from other studies that reported on the antibiofilm potential of AgNPs that were synthesized in different ways. For example, a study by [85] synthesized AgNPs from leaf extracts of *Allophylus cobbe* and tested their antibacterial and antibiofilm effects against *P. aeruginosa*, *E. coli*, and *S. aureus*. The results showed that AgNPs could disrupt the biofilm’s integrity, penetrate into the biofilm matrix, interfere with the quorum-sensing system, and enhance the efficacy of antibiotics. The authors of [86] synthesized AgNPs from leaf extracts of *Semecarpus anacardium*, *Glochidion lanceolarium*, and *Bridelia retusa* and evaluated their antibacterial and antibiofilm activities against different bacterial pathogens. The results demonstrated that AgNPs could attach to the biofilm surface, disrupt its structure, cause the leakage of EPS, and induce oxidative stress, DNA damage, protein dysfunction, and cell death. A study by [87] chemically synthesized AgNPs and characterized their effect on the biofilm formation and EPS production of *P. aeruginosa* and *S. aureus*. The results revealed that AgNPs could inhibit biofilm formation, reduce EPS production, and alter the morphology of biofilm cells. A study by [88] synthesized AgNPs using sodium borohydride as a reducing agent and examined their antibiofilm activity against extended-spectrum β-lactamases-producing *E. coli* and *Klebsiella* spp. The results indicated that AgNPs could prevent biofilm formation, detach biofilm cells, and increase the susceptibility of biofilm cells to antibiotics. A study by [89] synthesized AgNPs–chitosan composites and assessed their antibiofilm activity against multi-drug-resistant *Acinetobacter baumannii*. The results suggested that the AgNPs–chitosan composites could inhibit biofilm formation, disrupt biofilm structure, reduce biofilm biomass, and enhance the activity of antibiotics.

The anti-QS activity of AgNPs-S was an intriguing aspect of this study. The QS mechanism is a means of communication among bacteria that produces and detects the signal molecules responsible for regulating virulence factors [19]. AgNPs-S derived from Ajwa date saponins were found to inhibit QS in bacteria, thereby inhibiting progress in bacterial communication and virulence by interfering with QS. As a consequence of this finding, AgNPs-S are not only considered to be potential antimicrobial agents but also factors that could influence bacterial behavior and bacteria’s pathogenic capabilities.

An imbalance between the body’s protective antioxidant mechanisms and the generation of reactive oxygen species (ROS) leads to oxidative stress [90]. ROS, including free radicals, result in damage to cells, proteins, and DNA, leading to oxidative stress and the development of various diseases [91]. Natural compounds possess powerful antioxidant activity that plays a pivotal role in countering oxidative stress and maintaining cellular health by combating oxidative damage [92]. The antioxidant activity of AgNPs makes them intriguing therapeutic candidates. There are several mechanisms through which AgNPs can exhibit antioxidant activity. They accomplish this by donating electrons or accepting free radicals, one of the most important mechanisms through which they scavenge and neutralize reactive oxygen species (ROS). Aside from this, AgNPs can actively stimulate the activity of endogenous antioxidant enzymes, such as catalase, glutathione peroxidase, and superoxide dismutase (SOD), which assist in the reduction of oxidative stress and the conservation of cellular homeostasis [93,94,95]. The strong antioxidant activity of AgNPs-S was also demonstrated in the present study by their capacity to scavenge DPPH free radicals.

Lung cancer is one of the most important health issues worldwide; it is caused by the uncontrolled growth of abnormal cells in the lungs. The disease has a high mortality rate and limited treatment options, causing high mortality among various cancer-related deaths [96]. Many conventional treatment options, such as surgery, chemotherapy, and radiation therapy, have limited efficacy and can cause a range of side effects [97]. Thus, it is essential that novel approaches for the management and treatment of cancers such as lung cancer should be explored. Regarding possible therapeutic options, AgNPs are an emerging research area. Several potential advantages can be gained from using AgNPs to treat lung cancer. In terms of their cytotoxicity, AgNPs have been shown to selectively target cancer cells while sparing healthy cells [98]. The potential benefits of this targeted strategy could include the possibility of minimizing damage to healthy lung tissue, as well as reducing the adverse effects associated with conventional treatment methods [99]. Second, AgNPs have demonstrated promising cytotoxic properties. The process of apoptosis (programmed cell death) or autophagy (the self-destruction of cancer cells) is thought to be the mechanism by which these compounds induce cell death [100,101]. As well as inhibiting tumor angiogenesis, AgNPs exert antiangiogenic effects by forming new blood vessels to support tumor growth [102]. Furthermore, nanoparticles synthesized from silver can be used as drug carriers or to enhance the effectiveness of existing chemotherapeutics. Anticancer drugs can be loaded or functionalized onto them, enabling targeted delivery to the tumor site and potentially improving bioavailability. By using this approach, drug resistance can be overcome and treatments enhanced [103]. The results of our study showed a significant inhibition of the viability of human non-small-cell lung cancer cells after the action of AgNPs-S. Therefore, AgNPs-S show great promise as a novel treatment for lung cancer.

Overall, saponin-derived AgNPs from Ajwa dates possess significant antibacterial, antibiofilm, anti-QS, antioxidant, and cytotoxic potential. These nanoparticles demonstrate broad-spectrum antimicrobial activity, inhibit biofilm formation, interfere with QS, and exhibit antioxidant and cytotoxic effects against lung cancer cells. The multifaceted activities of AgNPs-S open up new avenues for their application in combating bacterial infections, biofilm-related complications, and cancer.

## 5. Conclusions

The development of saponin-derived silver nanoparticles from Ajwa dates represents a novel and promising approach in nanotechnology and biomedical research. The findings discussed in this study highlight the successful synthesis of AgNPs utilizing bioactive saponins found in Ajwa dates and emphasize their potential in many fields. The antibacterial potential of the novel AgNPs-S was evident in preventing the growth of both Gram-positive and Gram-negative bacteria. Moreover, the novel AgNPs-S exhibited significant antibiofilm activity by impeding the formation and growth of bacterial biofilms. The observed anti-QS activity of AgNPs-S provides a novel avenue for mitigating bacterial pathogenicity by disrupting QS signaling and downregulating virulent gene expression. Additionally, AgNPs-S demonstrated noteworthy antioxidant and cytotoxic effects, exerting cytotoxicity against lung cancer cells. Overall, this investigation into the antibacterial, antibiofilm, anti-QS, antioxidant, and cytotoxicity potential of saponin-derived AgNPs from Ajwa dates offers valuable insights into their multifunctional properties. The outcomes of this study hold great promise for the advancement of innovative bio-therapeutic approaches against bacterial infections, biofilm-related complications, and cancer. Continued research in this area may be a promising approach to reforming the field of nanomedicine and addressing the pressing challenges associated with antimicrobial resistance and cancer therapy.

## Data Availability

All data generated and analyzed during the course of this study are included in the article.
